# Maintained physical activity and physiotherapy in the management of distal arm pain: a randomised controlled trial

**DOI:** 10.1136/rmdopen-2018-000810

**Published:** 2019-03-04

**Authors:** Gareth T Jones, Gary J Macfarlane, Karen Walker-Bone, Kim Burton, Peter Heine, Candida McCabe, Paul McNamee, Alex McConnachie, Rachel Zhang, Daniel Whibley, Keith Palmer, David Coggon

**Affiliations:** 1 Epidemiology Group, Aberdeen Centre for Arthritis and Musculoskeletal Health, University of Aberdeen, Aberdeen, UK; 2 Arthritis Research UK/MRC Centre for Musculoskeletal Health and Work, University of Aberdeen, Aberdeen, UK; 3 MRC Lifecourse Epidemiology Unit, University of Southampton, Southampton, UK; 4 Arthritis Research UK/MRC Centre for Musculoskeletal Health and Work, University of Southampton, Southampton, UK; 5 Centre for Applied Research in Health, University of Huddersfield, Huddersfield, UK; 6 Warwick Clinical Trials Unit, University of Warwick, Coventry, UK; 7 Royal United Hospitals Bath NHS Foundation Trust, Bath, UK; 8 Nursing and Midwifery, University of the West of England, Bristol, UK; 9 Health Economics Research Unit, University of Aberdeen, Aberdeen, UK; 10 Robertson Centre for Biostatistics, University of Glasgow, Glasgow, UK

**Keywords:** fibromyalgis/pain syndromes, physcial therapy, health services research

## Abstract

**Objectives:**

The epidemiology of distal arm pain and back pain are similar. However, management differs considerably: for back pain, rest is discouraged, whereas patients with distal arm pain are commonly advised to rest and referred to physiotherapy. We hypothesised that remaining active would reduce long-term disability and that fast-track physiotherapy would be superior to physiotherapy after time on a waiting list.

**Methods:**

Adults referred to community-based physiotherapy with distal arm pain were randomised to: advice to remain active while awaiting physiotherapy (typically delivered after 6–8 weeks); advice to rest while awaiting physiotherapy, or immediate treatment. Intention-to-treat analysis determined whether the probability of recovery at 26 weeks was greater among the active advice group, compared with those advised to rest and/or among those receiving immediate versus usually timed physiotherapy.

**Results:**

538 of 1663 patients invited between February 2012 and February 2014 were randomised (active=178; rest=182; immediate physiotherapy=178). 81% provided primary outcome data, and complete recovery was reported by 60 (44%), 46 (32%) and 53 (35%). Those advised to rest experienced a lower probability of recovery (OR: 0.54; 95% CI 0.32 to 0.90) versus advice to remain active. However, there was no benefit of immediate physiotherapy (0.64; 95% CI 0.39 to 1.07).

**Conclusions:**

Among patients awaiting physiotherapy for distal arm pain, advice to remain active results in better 26-week functional outcome, compared with advice to rest. Also, immediate physiotherapy confers no additional benefit in terms of disability, compared with physiotherapy delivered after 6–8 weeks waiting time. These findings question current guidance for the management of distal arm pain.

**Key messages**

**What is already known about this subject?**

- The epidemiology and prognosis of distal arm pain and low back pain are similar. Management of the two conditions differs markedly – rest is not recommended for low back pain but is commonly advocated for persons with distal arm pain.
- We hypothesised that, in patients awaiting physiotherapy for distal arm pain, advice to remain active would result in superior functional recovery compared with advice to rest.

**What does this study add?**

- This is the first trial to compare the effectiveness of advice to maintain activity, versus advice to rest, among patients with distal arm pain. Advice to remain active results in better functional outcome at 26wks, compared with advice to rest.
- In addition, immediate physiotherapy confers no additional benefit in terms of disability, compared to physiotherapy delivered after 6-8wks.

**How might this impact on clinical practice?**

- The current study questions current guidance for the management of distal arm pain. These findings show that the ‘keep active’ management approach long advocated for low back pain has parallels in other regional musculoskeletal pain conditions.
- The current results also suggest that, for distal arm pain, early clinical intervention is not necessarily associated with improved outcome.

## Introduction

Upper limb pain is common among working-aged adults: one UK study demonstrated a 1-year period prevalence of pain lasting >1 day of 48%, among whom one-third had sought healthcare.^1^ The UK Health and Safety Executive estimated that 4 million working days were lost because of work-related upper limb disorders in Great Britain in 2016/2017.^2^ However, epidemiological investigations have been hampered by the lack of an agreed classification for upper limb disorders and the plethora of tautological terms implying causation including ‘repetitive strain injury’, ‘occupational cervicobrachial disorders’ and ‘work-related upper limb disorders’. More recently, with more consensus over case definitions,^3 4^ epidemiological studies of distal arm pain have found that both mechanical exposures (carrying weights, working with hands above shoulder height, bending/straightening the elbow, repetitive movements of the hand/wrist) and psychosocial factors (lack of job control, monotonous work, job dissatisfaction, negative beliefs, low expectation of recovery) were associated.^5 6^

Among those referred to physiotherapy with distal arm pain—that is, pain in the elbow, forearm, wrist or hand—around 50% still report pain at 1 year, with a substantial minority reporting that symptoms are unremitting.^7^ Interestingly, although conventional teaching would suggest that distal arm pain is caused by specific conditions (epicondylitis, tenosynovitis, de Quervain’s, carpal tunnel syndrome) or may be non-specific, there is little evidence of any difference in prognosis among these groups 1 year after physiotherapy referral.^7^

Thus, the evidence suggests that distal arm pain is common, disabling and has mechanical and psychosocial aetiology, and there is little evidence that separating states of ‘disease’ with different presumed causation or risk factors results in different therapeutic outcomes. In many respects, therefore, distal arm pain is similar to mechanical low back pain, the management of which was transformed when evidence emerged that bed rest was ineffective and that patients experienced improved outcomes if they maintained activities.^8^ Today, through education and large-scale health campaigns,^9^ practice has changed and unless there are red flags, few are sent for imaging, receive specific diagnoses or are advised to rest. In contrast, with the exception of specific treatments for certain underlying pathologies (eg, decompression for carpal tunnel syndrome), rest is the most prescribed recommendation in the management of distal arm pain, with the use of analgesics or anti-inflammatories as required, and referral for physiotherapy for persistent symptoms. Guidance from the UK National Health Service (NHS) website recommends that for tendonitis and tennis elbow, it is important to rest the injured limb and stop doing the exercise or activity that caused the symptoms,^10 11^ and the UK Health and Safety Executive advises that ‘if a task is causing or contributing to an upper limb disorder, the worker may need to stop doing that task for a time’.^12^ This guidance is based on the biomedical assumption that the tissues have been ‘injured’, and that the treatment of choice is therefore avoidance, even though there is no published evidence to support this assumption.

Written information providing evidence-based advice (The Back Book) is effective in promoting positive beliefs and contributing to improved clinical outcomes in back pain.^13 14^ It is plausible therefore that patients with distal arm pain could benefit from a similar approach. We conducted a randomised controlled trial to test the hypothesis that, among patients referred for physiotherapy with an episode of distal arm pain, advice to remain active and maintain usual participation results in a long-term reduction in disability, compared with advice to rest. Within the same trial, we also tested the hypothesis that, among patients referred for physiotherapy with an episode of distal arm pain, fast-track treatment would result in reduced long-term disability, as compared with treatment delivered after routine NHS waiting times.

## Methods

### Design

We conducted a multicentre, three-arm, randomised controlled trial. The study was registered on www.controlled-trials.com (reference: ISRCTN79085082) and its full protocol has been published.^15^ The study was approved by the UK South Central (Hampshire A) Research Ethics Committee (reference: 11/SC/0107).

### Patients

Participants were recruited from 14 NHS primary care physiotherapy referral centres, across the UK. Patients (aged ≥18 years) were potentially eligible for study if they were newly referred for outpatient physiotherapy with distal arm pain or disability. They were excluded, however, if they had received physiotherapy for distal arm pain within the past twelve months; the distal arm was not the main focus for treatment (eg, the pain was thought to originate from pathology in the neck); the pain was due to a fracture, systemic inflammatory disease, cancer or complex regional pain syndrome; symptoms were due to a specific disorder for which advice to remain active was contraindicated (eg, florid tenosynovitis); the appointment was classed as an emergency and/or they were involved in a legal dispute regarding their arm problem.

Potential participants were identified from outpatient clinic referrals and sent an invitation letter by a local research nurse, followed by a reminder if they failed to respond. Those who indicated that they might be willing to participate were invited to attend a pretrial screening visit to confirm their eligibility. At the visit, patients completed a questionnaire that asked about demographic characteristics, employment, symptom history, disability caused by the arm problem and other factors thought to be important potential prognostic markers or modifiers of treatment response. These included general health, physical and mental well-being, other symptoms (headache/abdominal pain/chronic widespread pain), somatic distress and health beliefs (especially fear avoidance). Patients also underwent a structured examination for the purposes of diagnosis and classification—the Southampton Examination Schedule for Upper Limb Disorders.^16^ This examination, involving inspection, palpation and clinical provocation tests, has previously been validated for outpatient and community settings and applied in large-scale epidemiological studies,^17^ and all research nurses were trained in the conduct of the examination by two of the investigators who originally developed and validated the examination schedule.

### Consent

Patients provided written informed consent before completing the baseline (screening) questionnaire and physical examination. They were also asked to give consent for randomisation should they prove to be eligible and this was reconfirmed, verbally, immediately prior to randomisation.

### Randomisation

Randomisation was conducted by the Robertson Centre for Biostatistics, University of Glasgow (part of the UK Clinical Research Collaboration registered Clinical Trials Unit) and was performed online, with telephone backup. Patients were allocated to one of the three treatment groups, using a mixed randomisation and minimisation algorithm to maintain treatment balance with respect to treatment centre, laterality (dominant, non-dominant, bilateral), a broad categorisation of diagnosis (predominant problem in the elbow vs wrist/hand) and baseline arm function, as assessed using a modified-Disabilities of the Arm, Shoulder and Hand (DASH) score (the primary outcome measure, see below, with scores grouped as 0–5, 6–8, or 9–11). One-third of patients were allocated completely at random, while two-thirds were allocated according to the minimisation algorithm. Randomisation to the three groups, or entry to the minimisation procedure, was determined according to a prespecified allocation schedule generated using the method of randomised permuted blocks of nine participants. Within the minimisation algorithm, any ties between treatment groups (ie, where allocation to more than one group would provide an equally low level of imbalance) were resolved by assigning the patient at random to one of the tied treatment groups. In terms of allocation concealment, as a consequence of their login permissions on the database, researchers from any one site were blind to data (and, thus, all randomisation information) from all other sites. Within each site, in order for an individual to guess which allocation was coming next, they would need to know the minimisation data for all previously allocated patients. Even then, they would be unable to determine whether the next patient was going to be allocated according to the minimisation algorithm or at random.

### Treatment

Participants were randomised to one of the following:

- Advice to remain active while awaiting usual care (waiting list) physiotherapy.
- Advice to rest while awaiting usual care (waiting list) physiotherapy.
- Immediate physiotherapy.

Participants randomised to advice to remain active received a seven-page booklet on how to deal with arm pain: ‘Keep Active to Recover Quickly’.^18^ The booklet was biopsychosocial in nature and developed from the findings of a contemporary Health and Safety Executive Research Report.^19^ It presented the core message that distal arm pain is common, lasting damage is rare and that recovery can be expected. In addition, it advocated a self-management approach with advice that early return to work and gradually increasing activity is helpful. The booklet was given to participants immediately post-randomisation, by the research nurse who conducted the screening/randomisation.

Participants randomised to advice to rest received a different booklet, designed to be similar in length and appearance. It was based on information available from the National Health Service at the start of the trial: ‘Advice and Guidance on Arm Pain—Causes, Diagnosis, Treatment*’*.^18^ It adopted a firmly biomedical approach, advocating rest and avoidance of activities that might further aggravate symptoms.

Participants randomised to immediate physiotherapy received an outpatient appointment at their earliest convenience. The trial was intended to be pragmatic in the delivery of physiotherapy, insofar as it reflected usual clinical care. However, to ensure that treatment programmes were compliant with both the Medical Research Council’s and the Consolidated Standards of Reporting Trials organisation’s guidance on developing complex interventions, we conducted a review of national and international treatment guidelines to ascertain current best practice. Therapists were presented with a summary of this review, but were able to treat patients on an individual basis with no restrictions on the number of appointments or treatment modalities. Thus, trial participation influenced *when* physiotherapy commenced, but not which specific therapeutic interventions were delivered.

Patients who were allocated to either of the advice groups were subsequently invited to attend physiotherapy, as per usual care, after a normal interval on a waiting list. At the start of the trial, this wait was approximately 6-8 weeks. Physiotherapists delivering their care received the same guidance on current best practice.

### Outcomes

Reflecting a move away from pain as the primary outcome in many pain trials, and the concept that function is a more meaningful end point, the primary outcome was a complete absence of disability at 26-weeks postrandomisation, as assessed using a modified version of the DASH questionnaire. The modified instrument was considered superior to the original for a number of reasons. For example, the original questionnaire contains no items that are specific to the distal arm, or that refer to activity limited specifically by pain. The modified instrument (mDASH) asked whether participants had experienced difficulty (yes/no/not applicable) with any of a list of 11 activities over the past 7 days, because of an ‘ache or pain in the elbow, forearm, wrist or hand’ and has previously been used in large scale epidemiological studies of distal arm pain.^7^

The mDASH was assessed by postal questionnaire 26 weeks after randomisation with postal reminders to non-responders. Outcome data were also collected, in the same manner, at 6 weeks and 13 weeks postrandomisation. Participants who did not return a questionnaire were contacted by telephone for verbal completion of the instrument.

Participants were also asked about behaviours and beliefs, so that the impact of advice could be assessed, and economic outcomes (costs associated with healthcare resource use including distal arm related hospital admissions, outpatient attendance and visits to/from relevant health professionals and health-related quality of life measured by EuroQol-5D and Short Form-12 questionnaires) were assessed.

### Sample size and statistical analysis

Previous studies have found that, among persons with distal arm pain receiving usual care (advice to rest, followed by physiotherapy), 51% reported being free of disability at 26 weeks.^7^ The current trial was powered on the assumption that, among participants who received advice to remain active followed by physiotherapy, this would increase to 70%. We required 148 subjects, per group, to detect this difference with 90% power at a 5% level of significance. From our experience of trials in other pain conditions,^20^ we anticipated a 20% loss to follow-up and, thus, we aimed to assign 185 patients per group. In addition, we aimed to allocate a further 185 patients to immediate physiotherapy, that is, a total of 555 randomised participants.

The primary analysis determined, at 26 weeks postrandomisation (1) whether participants who received advice to remain active were more likely to be free of disability, compared with those advised to rest and (2) whether those randomised to immediate physiotherapy were more likely to be free of disability, compared with those who received physiotherapy after a period on a waiting list. A mixed effects logistic regression model was used to estimate the OR for full recovery (mDASH=0) between different treatment groups. The model included treatment group (as a three-level categorical variable), age, gender, study centre, pain location (elbow, wrist/hand or both), laterality (dominant, non-dominant or bilateral) and baseline function (mDASH: 0–5, 6–8 or 9–11). The method of recycled predictions was used to estimate the absolute difference in the probability of being fully recovered between groups (advice to remain active vs advice to rest and immediate vs usually timed physiotherapy). The model used for the primary analysis was used to work out the predicted probability of the outcome for each individual in the study, based on the baseline characteristics of the individual as well as treatment allocation. Thus, we estimated the probability of full recovery for each individual in the study, under the assumption that all received a single treatment and compute the average (mean) predicted probability for the study population under this treatment. When repeated for each treatment group, this gives the overall probability of full recovery under each treatment (and the absolute differences between them). CIs were then computed using 1000 bootstrap samples.

All analyses were by intention-to-treat, and preplanned sensitivity analyses were conducted, making different assumptions about participants with missing data for the primary outcome: that all had fully recovered and that none had fully recovered.

A prespecified analysis was conducted to examine any evidence of heterogeneity of treatment effects, through the use of terms for interactions between treatment and each of the other variables in the regression models.^15^

Although the primary outcome treated the mDASH score dichotomously—that is, number of functional limitations at 26 weeks, none versus any—a preplanned secondary analysis considered the mDASH score as a continuous variable. A linear regression model was fitted, adjusting for the same factors as previously. This model was used to estimate the difference in mean mDASH scores between those randomised to receive advice to remain active and those advised to rest, and also those randomised to immediate physiotherapy and those randomised to usual care physiotherapy—for which the mean mDASH score was taken as the average of the two advice groups. Results were summarised as the mean differences in scores between the three treatment groups after adjustment for the covariates in the models.

Finally, we conducted a 26-week within-trial economic evaluation (cost-utility analysis, where benefits are measured in terms of quality-adjusted life years [QALYs]) from a UK health sector perspective. Assessments of the cost-effectiveness of alternative treatments are expressed as incremental cost-effectiveness ratios. Here, the health economic analysis will only be presented briefly, but full details of the economic evaluation methods and results are reported in a separate paper.^21^

## Results

**Figure 1:**
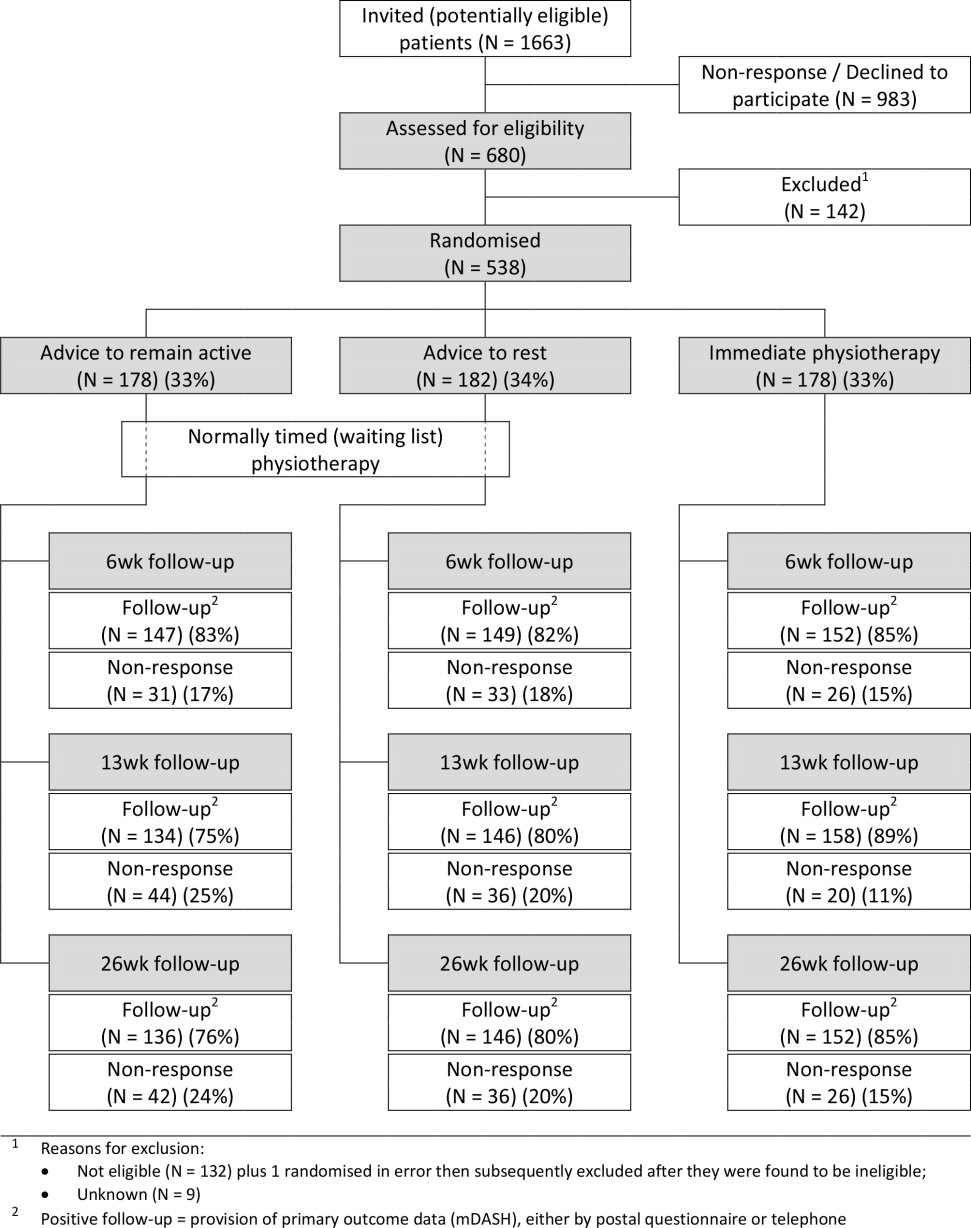
Consolidated Standards of Reporting Trials flow diagram.

Between February 2012 and February 2014, 1663 patients were invited to pretrial screening, of whom 680 were assessed for eligibility and 538 were randomised: 178 to advice to remain active, 182 to advice to rest and 178 advice to immediate physiotherapy; figure 1). The mean (SD) age of participants was 49 (14) years and 46% were male. Twenty-nine per cent of participants reported pain/disability in their elbow, 34% in their wrist/hand and 37% in both. According to the Southampton Examination Schedule, 67% had a specific disorder, while 33% had non-specific symptoms. Lateral epicondylitis, tenosynovitis and thumb osteoarthritis made up nearly two-thirds of the specific diagnoses recorded (23%, 21%, and 17%, respectively).Table 1 shows the demographic and baseline characteristics of the randomised participants, by treatment group.

**Table 1:** Baseline characteristics

Selected baseline characteristics*	Advice to remain active (N=178)*	Advice to rest (N=182)*	Immediate physiotherapy (N=178)*
Age, mean (SD)	49.1 (13.9) years	50.3 (14.2) years	48.2 (12.8) years
Gender (male)	77 (43.3%)	87 (47.8%)	81 (45.5%)
Body mass index, mean (SD)	27.3 (5.04) kg/m^2^	27.5 (5.25) kg/m^2^	27.1 (4.47) kg/m^2^
Employment status			
Full-time work	79 (44.4%)	94 (51.9%)	98 (55.4%)
Part-time work	39 (21.9%)	28 (15.5%)	29 (16.4%)
Retired	29 (16.3%)	31 (17.1%)	17 (9.6%)
Other	31 (17.4%)	28 (15.5%)	33 (18.6%)
Handedness			
Right	155 (87.1%)	154 (84.6%)	162 (91.0%)
Left	18 (10.1%)	17 (9.3%)	13 (7.3%)
Both	5 (2.8%)	11 (6.0%)	3 (1.7%)
Broad diagnosis			
Elbow	50 (28.1%)	53 (29.1%)	53 (29.8%)
Wrist/hand	61 (34.3%)	62 (34.1%)	58 (32.6%)
Both	67 (37.6%)	67 (36.8%)	67 (37.6%)
Specific problem†			
Specific	127 (74.7%)	107 (60.5%)	114 (66.3%)
Non-specific	43 (25.3%)	70 (39.5%)	58 (33.7%)
Laterality of problem			
Dominant	80 (44.9%)	83 (45.6%)	81 (45.5%)
Non-dominant	54 (30.3%)	55 (30.2%)	52 (29.2%)
Bilateral	44 (24.7%)	44 (24.2%)	45 (25.3%)
Duration of problem			
≤1 month	38 (22.2%)	38 (21.6%)	26 (15.2%)
>1 month	133 (77.8%)	138 (78.4%)	145 (84.8%)
Pain severity, median (IQR)‡			
Right side	5 (1, 7)	5 (2, 7)	5 (2, 7)
Left side	3 (0, 6)	3 (0, 6)	3 (0, 7)
Tampa Scale of Kinesiophobia, mean (SD)	3.62 (5.60)	3.67 (5.77)	3.63 (5.99)
Baseline mDASH‡ score, mean (SD)	5.9 (2.8)	5.8 (2.8)	5.9 (2.7)
EQ-5D health utility score, mean (SD)	0.674 (0.222)	0.667 (0.233)	0.655 (0.224)

*All data presented as N (%), unless otherwise specified.

†Numbers do not sum to randomised totals, due to missing data from clinical examination and, thus, an inability to classify all participants.

‡On how many days in the past 7 days did you have pain in your elbow, forearm, wrist or hand? (0–10 numerical rating scale).

EQ-5D, EuroQol 5D; mDASH, modified Disabilities of Arm Shoulder and Hand questionnaire.

**Figure 2:**
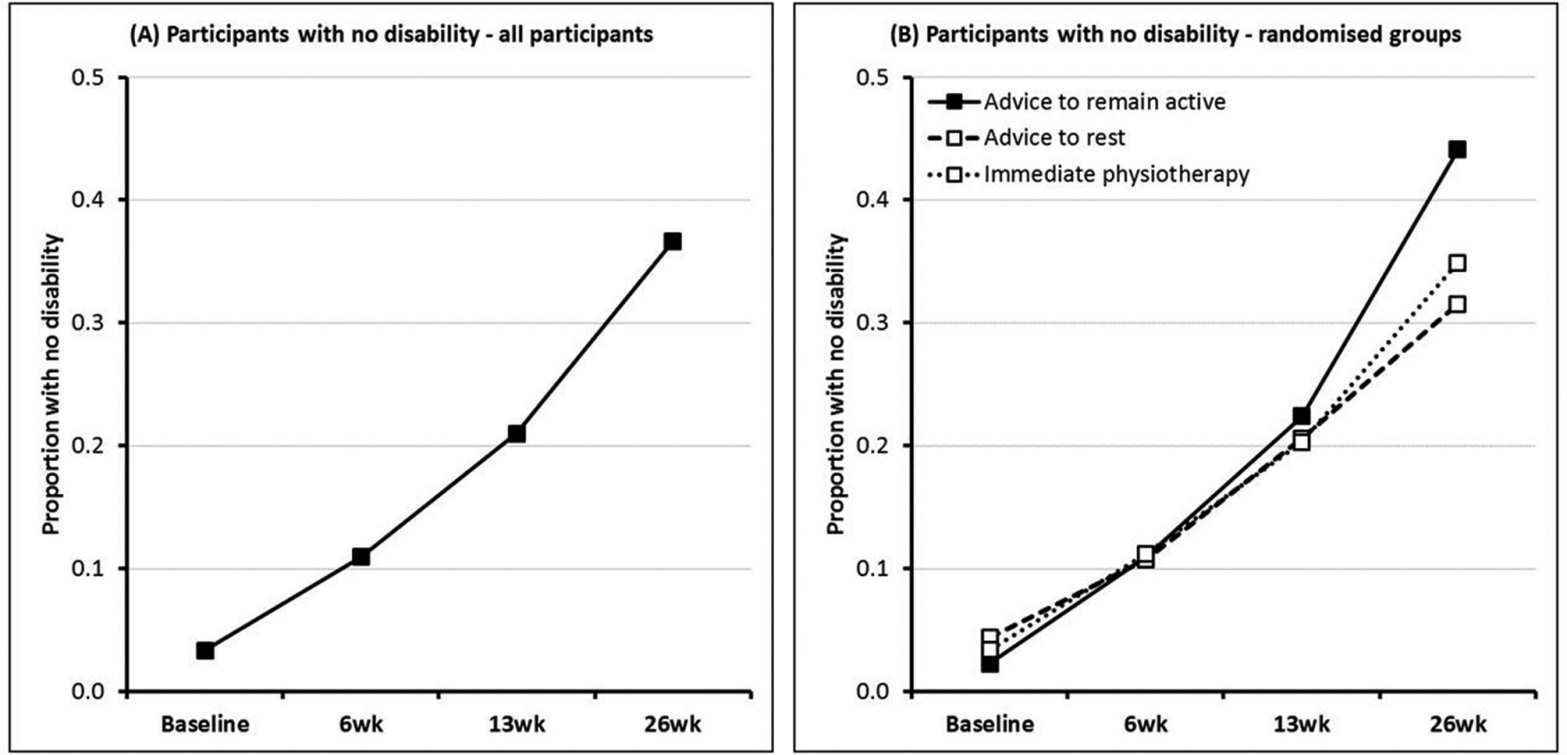
Proportion of responders with no disability, at each time point.

At 26 weeks, 81% of participants provided primary outcome data (figure 1) with similar proportions between treatment groups: 136 (76%), 146 (80%) and 152 (85%) in the advice to remain active, advice to rest and immediate physiotherapy groups, respectively, of whom 60 (44%), 46 (32%) and 53 (35%) reported full recovery. The figure 2 (panel A), illustrates the proportion of respondents at each time point with no disability (mDASH=0). This increased from 3.3% at baseline to 37% at 26 weeks. The figure 2 (panel B) shows the prevalence of complete recovery in the three treatment groups at different stages of follow-up. Differences at 6 weeks and 13 weeks were small, but at 26 weeks, the probability of full recovery was 45.1% among participants who received advice to remain active, compared with 32.2% among those who received advice to rest; a difference of 12.9% (95% CI 2.3% to 23.7%) (table 2). This equates to an OR for full recovery at 26 weeks of 0.54 (95% CI 0.32 to 0.90) for advice to rest versus advice to remain active. In contrast, there was no significant difference in the probability of full recovery at 26 weeks between participants randomised to immediate (35.8%) as compared with routinely timed physiotherapy (38.6%), a difference of −2.8% (95% CI −11.3% to 6.5%). The adjusted OR for full recovery at 26 weeks was 0.64 (95% CI 0.39 to 1.07) for immediate physiotherapy versus advice to remain active. Sensitivity analysis, making different (prespecified) assumptions about the prevalence of full recovery at 26 weeks and, separately, excluding participants who provided follow-up data by telephone, did not alter these conclusions (table 2).

**Table 2:** Probability of full recovery at 26 weeks

	Probability of full recovery at 26 weeks (95% CI)			
	Main analysis	Sensitivity analysis*	Sensitivity analysis^†^	Sensitivity analysis‡
Advice to remain active	45.1% (37.3 to 53.0)	58.7% (51.9 to 66.0)	34.4% (27.7 to 41.5)	44.6% (34.1 to 54.8)
Advice to rest	32.2% (24.4 to 39.7)	45.6% (37.8 to 52.9)	25.2% (18.4 to 31.7)	23.7% (14.3 to 33.1)
Difference	12.9% (2.3 to 23.7)	13.1% (3.4 to 22.3)	9.2% (0.3 to 18.5)	21.0% (7.6 to 34.7)
Immediate physiotherapy	35.8% (28.7 to 42.9)	44.7% (37.8 to 51.4)	29.6% (23.1 to 36.2)	33.2% (24.8 to 42.7)
Normally timed physiotherapy	38.6% (32.8 to 44.2)	52.1% (46.7 to 57.7)	29.8% (24.3 to 34.8)	34.1% (26.9 to 41.3)
Difference	−2.8% (−11.3 to 6.5)	−7.5% (−16.1 to 0.7)	−0.2% (−8.3 to 8.1)	−0.9% (−11.9% to 10.4)

*Assumes all participants with missing data at 26 weeks were fully recovered.

†Assumes no participants with missing data at 26 weeks were fully recovered.

‡Restricting analysis to follow-up questionnaire respondents only.

There was no clear evidence of an effect in the early part (≤13 weeks) of the follow-up period from advice to remain active (figure 2). The differences in the probability of full recovery at 6 weeks and 13 weeks, in comparison with those who received advice to rest, were 0.3% (95% CI −6.4% to 6.8%) and 3.1% (95% CI −6.7% to 12.0%), respectively (table 3). Similarly, there was no evidence of short-term benefit from immediate physiotherapy versus usually timed treatment, differences in the probability of full recovery being −0.4% (95% CI −6.4% to 5.3%) and −1.6% (95% CI −9.5% to 5.9%) at 6 weeks and 13 weeks, respectively. While patients were more than five times more likely to report full recovery at 26 weeks than 6 weeks (OR 5.31; 95% CI 3.86 to 7.31) there was no evidence of a time-by-treatment interaction (p=0.750).

**Table 3:** Probability of full recovery, at 6 weeks and 13 weeks

	Probability of full recovery (95% CI)	
	6weeks	13weeks
Advice to remain active	10.4% (5.7 to 15.2)	22.8% (16.3 to 30.0)
Advice to rest	10.1% (5.0 to 15.3)	19.7% (13.0 to 26.3)
Difference	0.3% (−6.4 to 6.8)	3.1% (−6.7 to 12.0)
Immediate physiotherapy	9.8% (4.9 to 14.9)	19.6% (13.5 to 26.0)
Normally timed physiotherapy	10.3% (6.7 to 14.0)	21.2% (16.2 to 26.0)
Difference	−0.4% (−6.4 to 5.3)	−1.6% (−9.5 to 5.9)

**Figure 3:**
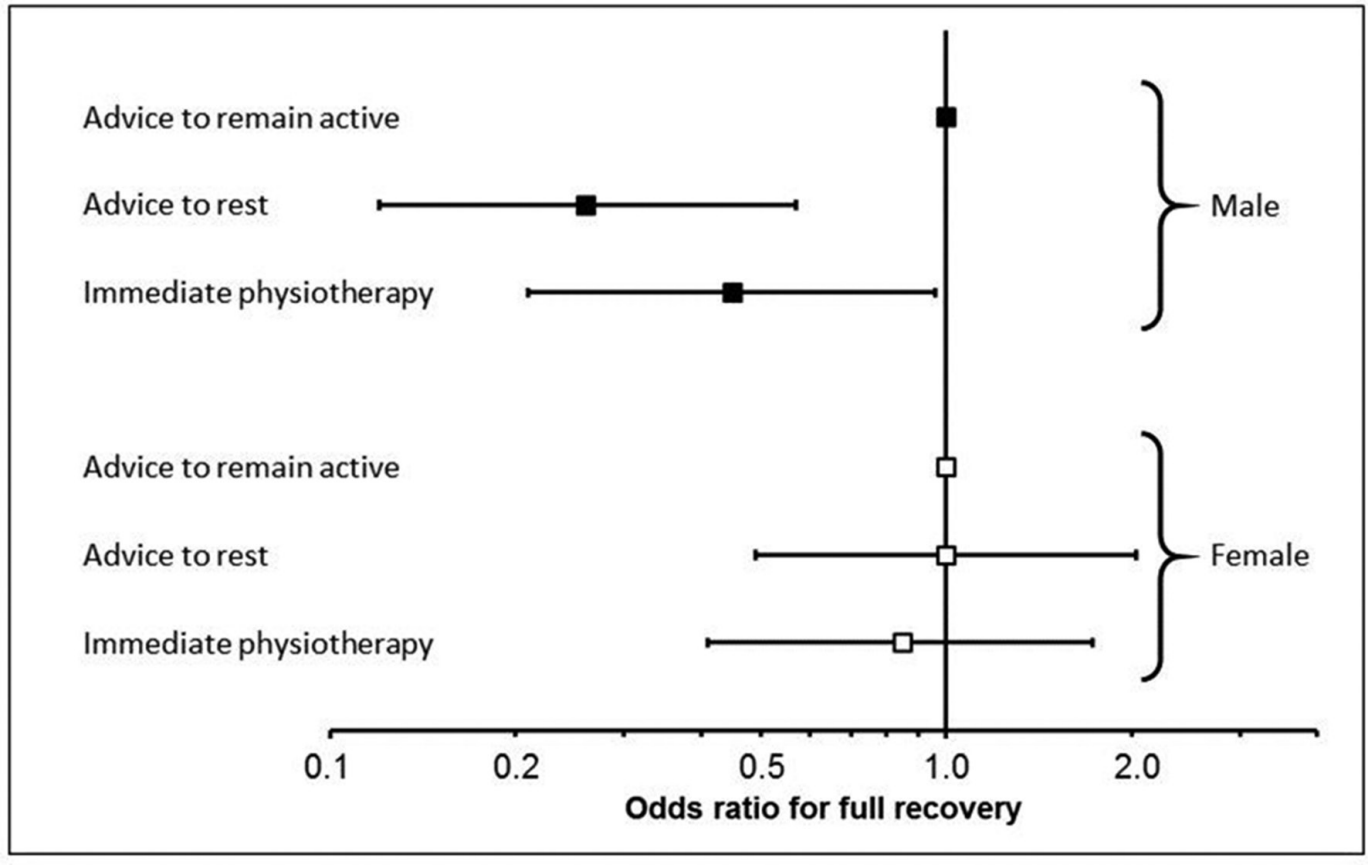
Impact of treatment on full recovery at 26 weeks by gender.

There were indications of a gender-by-treatment interaction (p=0.047) such that, compared with men randomised to advice to remain active, those randomised to either of the other two groups were less likely to report full recovery at 26 weeks, whereas this did not apply in women (figure 3). Although there were other minor differences between subgroups, there were no other large or statistically significant interactions between treatment group and the other variables in the final model.

While the proportion of participants reporting full recovery at 26 weeks differed between treatment groups, no significant difference was observed in mean mDASH score between those who received advice to remain active and those advised to rest (−0.50; 95% CI −1.13 to 0.13) or between those who received immediate as compared with normally timed physiotherapy (0.24; 95% CI −0.29 to 0.78).

### Cost-effectiveness

The number of treatment sessions was similar between treatment groups (table 4), indeed the difference in mean NHS costs between the three groups was small and not statistically significant. Adjusted mean cost differences were £88 (95% CI −£14 to £201) for advice to remain active versus advice to rest and −£14 (95% CI −£87 to £66) for immediate versus normally timed physiotherapy (table 5). The differences in mean QALYs were also similar between treatment groups: adjusted mean QALY differences were 0.0095 (95% CI −0.0140 to 0.0344) for advice to remain active vs advice to rest and 0.0143 (95% CI −0.0077 to 0.0354) for −£14 (95% CI −£87 to £66) for immediate versus normally timed physiotherapy.

**Table 4:** Number of physiotherapy treatment sessions, per group

	Median	IQR	Range
Advice to remain active	3	2–4	1–14
Advice to rest	3	2–5	1–15
Immediate physiotherapy	3	2–5	1–12

**Table 5:** Mean overall costs and QALYS for the three treatment groups (over 26 weeks)

	Mean (SD) NHS costs	Mean (SD) QALYs	Incremental mean costs (95% CI)*	Incremental mean QALYs (95% CI)*	Mean ICER† (£/QALY)
Group1 Advice to remain active	£309.91 (£321.45)	0.372 (0.111)	−£87.87 (−£14.33 to £200.83)	0.0095 (−0.0140 to 0.0344)	Group 1 versus Group 2: £9256
Group 2 Advice to rest	£223.15 (£225.39)	0.366 (0.077)	–	–	–
Group 3 Immediate physiotherapy	£221.46 (£220.54)	0.388 (0.089)	−£14.22 (−£87.14 to £66.01)	0.0143 (−0.0077 to 0.0354)	Group 3 versus Group 1 and 2 Group 3 dominant (cost per QALY gained <0)

*Adjusted for age, gender, work status, modified-DASH, EQ-5D health utility score and NHS cost and bootstrapped non-parametric 95% CI.

†Mean ICER, adjusted for age, gender, work status, modified-DASH, EQ-5D health utility score and NHS cost.

DASH, Disabilities of the Arm, Shoulder and Hand; EQ-5D, EuroQol-5D; ICER, incremental cost-effectiveness ratio; NHS, National Health Service; QALY, quality-adjusted life year.

## Discussion

Among patients referred to physiotherapy with distal arm pain, we have demonstrated that advice to remain active is associated with better functional recovery at 26 weeks, compared with advice to rest. In addition, we have shown that physiotherapy delivered immediately offers no additional benefit in terms of disability at 26 weeks, compared with physiotherapy delivered after a 6–8 weeks waiting time.

Several methodological issues should be considered when interpreting these findings. First, we expected that approximately half of participants would be symptom-free after 26 weeks, based on a previous study of distal arm patients recruited from primary care and physiotherapy.^7^ In fact, according to the primary outcome chosen for this study (complete absence of disability (mDASH=0) after 26 weeks), the proportion of patients in any of the three arms who had fully recovered was lower than 50%. The original power calculation anticipated a 51% recovery rate in the group advised to rest. The primary analysis model predicted the recovery rate in this group to be 32.2%; thus, the original target of 148 per group had 90% power to detect an increase to 51.5%. This absolute increase in recovery of 19.3% is comparable to the original sample size calculation, which gave 90% power to detect an absolute increase in recovery of 19% (from 51% to 70%). Given the achieved sample size for the primary analysis (N=136 and N=146 in the active and rest groups, respectively), the study had 89% to detect an increase in recovery from 32.2% to 51.5%. Therefore, we do not believe that our study was underpowered to detect important differences between groups.

Second, although we conducted intention to treat analysis, it is important to note that primary outcome data could not be obtained for 19% of participants. Importantly, sensitivity analyses, which assumed both of the extreme scenarios—that all or none of those with missing data had fully recovered—indicated that the main trial results were not critically influenced by the unavailable data.

Although patients were potentially eligible if they were referred to physiotherapy with pain and/or disability in the distal arm, referral letters were not available, and it is not known how many participants were referred with pain, disability or both. However, at the point of randomisation, 458 (85%) reported pain and disability; 62 (12%) disability but no pain; 17 (3%) pain but no disability and one neither (but was randomised nonetheless, because the eligibility criteria applied at screening and initial invitation). Treatment allocation was similarly balanced between these four groups and, in the two groups reporting disability at baseline, the proportion achieving the primary outcome was comparable: 36% and 32%, respectively. Interestingly, of those without disability at baseline, around one-quarter had disability at 26 weeks.

Follow-up questionnaires were required at 6 weeks, 12 weeks and 26 weeks. However, anticipating some non-response, we also obtained ethical approval to contact non-responders (after a postal reminder) by telephone to collect more limited follow-up data. Our follow-up response rates were much as expected, but over 26 weeks, there was an increase in the proportion of responders who provided outcome data by telephone, with small (although non-significant) differences between groups (figure 1). Importantly, post hoc analysis, restricted to questionnaire respondents, revealed similar findings to the main analysis (table 2) and did not call into question the validity of conclusions based on the main analysis.

The prestated outcome was complete recovery from disability^15^ and with this outcome advice to remain active was clearly superior to advice to rest. However, when the mDASH was analysed as a continuous variable, the difference between the advice groups, although in the same direction, was not statistically significant (difference in mean mDASH score between active vs rest advice groups: −0.50 (95% CI −1.13 to 0.13). One can speculate the reasons for this, although it may be that mean values for the mDASH were strongly influenced by a small minority of participants with relatively high scores, making it harder to demonstrate statistical significance.

This study was predicated on the assumption that distal arm pain might be similar to mechanical low back pain—both are common and disabling, have similar profiles of risk factors with little evidence that, among those referred for physiotherapy, separating different ‘diseases’ leads to any difference in prognosis.^22^ The superiority of avoiding rest in back pain management was first demonstrated >20 years ago, but rest continues to be widely advocated in the immediate management of distal arm pain under the beliefs of a biomechanical causation.^10 11^ Our results provide evidence that, among this group of patients, as for back pain, advice to rest may not be the best approach. The content of the experimental leaflet gave reassurance, explained that often there is no obvious cause of injury, promoted the maintenance of activity and advised early return to work. Our study could not inform as to whether the advice leaflets were read or not. However, it is interesting to note that at 6 weeks postrandomisation, 50.5% of those in the ‘active’ advice group reported that they were trying to be more active than when their pain had first started, compared with only 29.9% of those advised to rest (difference: 20.6%; 95% CI 8.0% to 33.1%). This differential was maintained at 13 weeks (47% vs 32%), but was no longer apparent at 26 weeks (49% vs 45%). Although not conclusive, this is consistent with the hypothesis that the positive outcome seen at 26 weeks was built on early changes in health behaviour and/or associated health beliefs. Alternatively, it may be that keeping active during the initial waiting list period enables participants to derive more benefit from physiotherapy when they receive it. This would also explain the fact that the divergence in effect is seen at 13 weeks (ie, after advice and after physiotherapy). It would have been interesting to have objectively measured activity levels, but this was not feasible in the current study. It would be naive to assume that all participants adhered perfectly to the advice they were given. However, lack of adherence would tend to bias the findings towards the null hypothesis, that is, more likely to find no differences between groups, and makes the fact that a difference was observed even more remarkable.

A small proportion of participants in the advice groups sought private physiotherapy to obtain treatment sooner than was available via the NHS. If the proportion of patients doing this was greater among those advised to remain active, it might explain their better outcomes. However, a comparison showed that the rates of private physiotherapy were similar between advice groups (12% and 15%) and therefore unlikely to explain the findings. Additionally, in confirmatory analyses in which those who took up private physiotherapy were excluded, the trial results were unaltered.

Because physiotherapy regimens were not specified in the study protocol, it might be feasible that there had been some systematic differences in the treatment given between groups, perhaps in the number of sessions or the treatment modalities employed. However, these data were recorded throughout the trial and we found no differences in the median number of treatment sessions between groups. While the frequency of physiotherapy sessions varied depending on centre and therapist, most participants were treated once per week until discharge, as one would expect in usual care—reflecting the pragmatic nature of the trial. A comparison of treatment modalities between those randomised to immediate or ‘waiting list’ physiotherapy revealed that those in the ‘immediate’ groups were more likely to receive Protection, Rest, Ice, Compression and Elevation (PRICE), but this is to be expected since PRICE is recommended in response to acute pain. Indeed, the ‘immediate’ group, possibly as part of ‘Protection’, were more likely to be given an orthotic device. In interpreting these data, however, there is no reason to expect that greater use of these modalities would detract from the benefits of early physiotherapy, and there were no other large, consistent, or statistically significant differences in treatment modalities between any of the three groups. For all groups, education and exercise were the most commonly recommended therapies.

Most participants had experienced symptoms for >1 month before recruitment. It is perhaps not surprising, therefore, that differences between the two advice groups did not emerge immediately, but rather were gradual, and only statistically significant at 26 weeks (the prespecified primary end point). Although the difference was not great, a slightly smaller proportion of patients in the immediate group presented with a short duration of symptoms (≤1 month): 15% versus 22% in each of the advice groups, but, symptom duration was not associated with prognosis. We also found no evidence of heterogeneity of the effect of advice to remain active between participants with a specific versus non-specific diagnosis. This supports previous epidemiological work suggesting no difference in prognosis between those with specific/non-specific symptoms.^7^

The interaction between gender and treatment is intriguing. This (prespecified) analysis was conducted to determine whether there was any evidence of heterogeneity of treatment effect between treatment group and other variables in the final regression model, rather than because of any particular a priori clinical hypothesis. Women, at baseline, reported higher levels of disability than men and the probability of full recovery was inversely proportional to baseline disability. It may be, therefore, that it was simply harder for them to reach full recovery after 26 weeks than it was for men. However, the explanation for this finding cannot be determined from the available data.

In summary, pain and disability in the distal arm is common. Although aetiological and prognostic factors are similar to those for low back pain, hitherto, its management has been very different. Rest is rarely advised for back pain but is commonly recommended for patients with distal arm pain, despite the lack of any published evidence to support this management strategy. Our trial calls this approach into question and provides the first evidence that advice to remain active is associated with superior clinical outcomes. Furthermore, we found no indication that earlier initiation of physiotherapy improved long-term clinical outcome. This has important implications for employers and their occupational health advisors, as well as for those working in primary care and physiotherapy. Although immediate physiotherapy was shown to be no more effective than physiotherapy delivered after a waiting list, it was no more costly. What is clear, however, is that the early management should encourage activity: this is more effective, in terms of 6 month outcome, and also more likely to be cost-effective than advice to rest. Undoubtedly, further independent confirmation would strengthen our conclusions but there is unlikely to be additional randomised controlled trial data available. Therefore, based on the previous epidemiological data, an extensive evidence base about back pain, and from the current trial results, we recommend that, for the majority of cases, the most sensible course of action would be to stop advising that patients with distal arm pain rest, while awaiting physiotherapy.
